# Information and Empowerment of Families of Children With Cerebral Palsy in Brazil: The Knowledge Translation Role of Nossa Casa Institute

**DOI:** 10.3389/fresc.2021.709983

**Published:** 2021-07-19

**Authors:** Marina J. Airoldi, Beatriz S. Vieira, Rachel Teplicky, Deborah Chalfun, Rafael G. A. S. Bonfim, Marisa C. Mancini, Peter Rosenbaum, Marina B. Brandão

**Affiliations:** ^1^Nossa Casa Institute, Campinas, Brazil; ^2^CanChild Centre for Childhood Disability, Hamilton, ON, Canada; ^3^Occupational Therapy Department and Graduate Program in Rehabilitation Sciences, Universidade Federal de Minas Gerais, Belo Horizonte, Brazil

**Keywords:** knowledge translation, family-centered, action cycle, pediatric rehabilitation, cerebral palsy

## Abstract

Knowledge translation (KT) is gaining attention in the pediatric rehabilitation field. *Nossa Casa Institute* is the first organization in Brazil aiming to foster cerebral palsy (CP) awareness and empower families by discussing reliable information. This study aims to build a network where individuals with CP and their families, researchers, health care professionals, and services can communicate and share experiences. In this article, we describe the experience of planning and conducting an educational and interactive online workshop to foster principles of family-centered service (FCS). We used the action cycle from the Knowledge to Action (KTA) framework to describe and ground the proposed activities. In Module 1, “Challenges and barriers to incorporate family-centered principles,” we discussed the historical perspective, main principles, and challenges related to FCS implementation. Module 2, “What is my contribution to the family-centered service?” was aimed to foster strategies to improve the implementation of principles of FCS in the care of children with disabilities. In Module 3, “What can we do together?” the groups presented their ideas and suggestions. This interactive and educational workshop was an opportunity for *Nossa Casa Institute* to disseminate accessible and reliable information regarding FCS and to empower families to participate actively in the rehabilitation process and advocate for the best provision of care for their children. Future actions of *Nossa Casa Institute* include the coordination of a national conference to connect families, individuals with CP, healthcare and rehabilitation professionals, and researchers. There is also a need, and opportunity, for formal evaluation of these KT activities.

## Introduction

Knowledge translation (KT) is defined by the Canadian Institutes for Health Research as “a dynamic and iterative process that includes synthesis, dissemination, exchange, and ethically sound application of knowledge” (1). KT aims to diminish the gap between the research literature and its application into practice (2–4). In this process, there should be an articulation between researchers and the end-use stakeholders (i.e., health care professionals, policymakers, and patients) (4–8).

Different models have been proposed to explain the KT process (9). The Knowledge to Action Model (KTA) proposed by Graham et al. (2) has been extensively used in health care studies (10). In the KTA model, KT involves two main elements: knowledge creation and an action cycle (2). Knowledge creation involves knowledge inquiry, synthesis, and the formation of tools and products. The action cycle is composed of activities to allow knowledge application. It encompasses identification of the problem to be addressed, adaptation of the knowledge to the local context, assessments of barriers to knowledge use, selection and implementation of interventions, and monitoring, evaluating, and sustaining knowledge use (2).

Knowledge translation is gaining considerable attention in the rehabilitation field (6–8, 11, 12). The resources and strategies used in KT vary from the provision of single activities (e.g., educational workshops) to multifaceted approaches, with the use of active and multiple tools (e.g., educational workshops and public audit) (6, 11). In pediatric rehabilitation, most initiatives are tailored to overcome the knowledge-to-practice gap, with specific actions directed to health care professionals and services, aimed to promote changes in clinical behavior and to improve care (11, 13–15). Recently, some KT activities with the participation of families have been reported (7, 15, 16).

CanChild Centre for Childhood Disability Research is a university-based health services research program, and an example of an organization pursuing research, education, and KT in pediatric rehabilitation (15–17). The center's interest and engagement with KT have become a major focus of its activities, grounded in the principles of the KTA framework outlined above.

Despite progressive efforts to promote and achieve KT actions in health, many challenges are reported (10, 18–20). Ferraz et al. (18), in a scoping review, synthesized the main challenges. One of the challenges is the lack of cohesion among researchers, populations, and health care policymakers. In this sense, researchers and end-user stakeholders should communicate, so that research questions and methods meet the needs of the community as identified by the community. Second, is the difficulty for health professionals to translate and apply new knowledge. This may be due to the lack of abilities of clinicians or time available to appraise the literature critically and to understand statistical methods. Third, the lack of incentives and supports from health institutions to engage in KT restricts opportunities for continued education of their professionals (18).

Challenges for KT in health are intensified in developing countries (20–22). To the best of our knowledge, there was no structured program in KT in the pediatric rehabilitation field in Brazil before the foundation of *Nossa Casa Institute* in 2016. It is the first online platform to discuss the daily living of individuals with cerebral palsy (CP) and their families, considering the importance to facilitate both communication and implementation of knowledge about CP among all stakeholders in Brazil.

## Nossa Casa Institute

*Nossa Casa Institute* is a non-profit organization, funded and started in 2016, that grew out of collaboration among individuals with CP, families, health care professionals, and researchers. The meaning of “*Nossa Casa*” in English is “*Our Home*.” These words were chosen to represent a place for open discussion of information and ideas about CP in a welcoming, friendly, and safe way. This institute aims to build a network where local and international researchers, health care professionals and services, individuals with CP, and their families can communicate, discuss, and share experiences. In 2018, *Nossa Casa Institute* was recognized by the Cerebral Palsy Foundation in the World Cerebral Palsy Day, winning the “Public Awareness” award.

The online tools of *Nossa Casa Institute* include a website, an Instagram account with almost 16,000 followers, a Facebook fan page with 20,800 participants, and 2,500 subscribers on its YouTube account. As a KT Institute, we seek to discuss reliable information regarding issues in the daily lives of people with CP and evidence-based information about assessment and intervention strategies for this population, using plain, accessible user-friendly language. Four groups of activities are conducted by *Nossa Casa Institute:* (1) online interactions, including social media posts and live sessions; (2) development of educational and informative materials, such as videos, awareness campaigns, tutorials, and translated guidelines and worksheets; (3) educational training, workshops and conferences, with national and international experts discussing topics related to CP; and (4) collaboration with researchers to conduct studies aimed at promoting the daily functioning of individuals with CP. Table 1 shows the main actions developed by *Nossa Casa Institute* since its foundation.

**Table 1:** Description of main actions and activities developed by *Nossa Casa Institute*, from 2016 to 2021.

**Main actions**	**Activities**
Online interactions, including social media posts and live interactions	463 Instagram Posts ( @nossacasa.org.br) 40 Instagram lives ( @nossacasa.org.br) 57 Youtube videos (Instituto Nossa Casa) Facebook fanpage 1 website (www.nossacasa.org.br)
Development of educational and informative materials regarding individuals with cerebral palsy (CP), such as videos, tutorials, and translated guidelines and worksheets	8 video animations: - “Let's talk about cerebral palsy!”, 2,900 views (Available at: https://youtu.be/0qm142gZ3Hc) - “What's cerebral palsy?” 52,400 views (Available at: https://youtu.be/oo4NIPgqLW4) - “F-Words,” 10,000 views (Available at: https://youtu.be/xPMzPJwWop8) - “Perinatal stroke,” 6,800 views (Available at: https://youtu.be/ml5rEOTUImg) - “Early intervention in hemiparesis,” 15,800 views (Available at: https://youtu.be/MpSCPKHVDmw) - “Cerebral Palsy and ICF,” 6,900 views (Available at: https://youtu.be/JknMCYaopF8) - “What is evidence-based practice?”, 4,700 views (Available at: https://youtu.be/aNck3M5QWqo) - “Family-Centred Service” (to be released in 2021) 2 video tutorials: - “Moving is Power!” (adapted from Go Baby Go) (to be released in 2021) -Low-cost adapters for play (to be released in 2021) -Translation: FCS worksheets from Canchild (to be released in 2021)
Educational training, workshops, and conferences, with national and international experts	- “General Movements Assessment Workshop,” conducted by Christa Einspieler, Campinas, Brazil, 2016 - World CP Day Campaign in Brazil (2017, 2018, 2019, 2020) - “Children with CP GMFCS levels IV and V: what should we do?”, conducted by Ginny Paleg, Campinas, Brazil, 2018 - International Cerebral Palsy Conference (800 participants, 35 speakers), Campinas, Brazil, 2019 - “Moving is Power!”, conducted by Marina Alroldi and Beatriz Vieira, 200 participants, online event - “Family-Centred Workshop,” conducted by Marina Brandão, Peter Rosenbaum, Rachel Teplicky, 86 participants, online event, 2020 - “Cerebral Palsy Online Congress„” 1,700 participants, 72 speakers, online event, 2021
Collaboration with researchers and institutions	- CanChild Centre for Childhood Disability, Hamilton, Ontario, Canada -Universidade Federal de Minas Gerais, Belo Horizonte, Brazil

The activities at *Nossa Casa Institute* involve active collaboration among families, individuals with CP, health care professionals, and researchers. Families and individuals with CP participate in online interactions, suggest themes to be discussed, conduct live interactions, and review the content of the shared posts. As for the educational and informative materials, families and individuals with CP collaborate with the conception, illustration, and description of the videos and with content review. Educational training was originally directed to health care professionals and researchers (e.g., General Movement Assessment workshop). In 2019, the International CP Conference was opened to families who showed interest in attending the event. At this conference, one speaker presented his experience as an adult with CP and two mothers of a child with CP spoke. Nowadays, the main efforts of *Nossa Casa Institute* are focused on planning and developing educational training activities with accessible language and active participation of families and individuals with CP. Main representatives of families and individuals with CP are also collaborators of research initiatives in KT at *Nossa Casa Institute*, including the conception of the studies, data collection, and writing process.

## Family-Centered Workshop: From Challenges to Shared Solutions to Implement Family-Centered Services (FCS)

The active participation of families in the rehabilitation of their children with disabilities is widely encouraged in the literature (23–26). To make the best decisions for their children, families should have access to reliable and accessible information and the opportunity to express their preferences, interests, and concerns (23, 24, 27). Although literature on FCS has been available since the 1990s, the implementation of its principles in practice is still challenging. Thus, the proposal of an FCS Workshop with online interactions and educational material in Portuguese (Brazil) aimed to facilitate the incorporation of FCS principles in the pediatric rehabilitation field.

**Figure 1:**
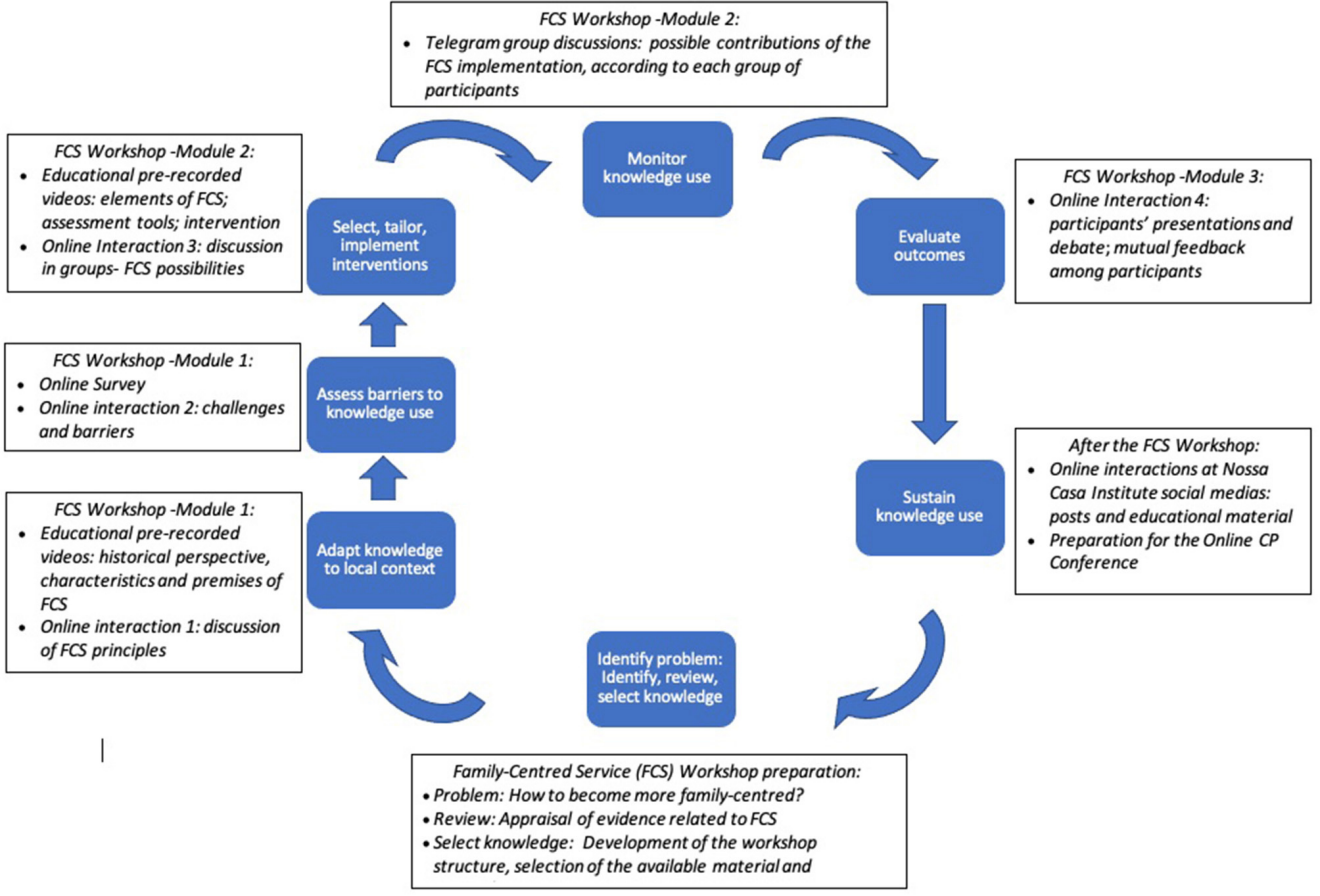
Illustration of the FCS Workshop based on the action cycle from the Knowledge to Action (KTA) model [adapted from Graham et al. (2)].

In this “perspective” article, we describe the FCS Workshop to illustrate the role of the *Nossa Casa Institute* in KT activities. We anchored the activities of this workshop in the action cycle of the KTA framework (2) (Figure 1).

### Identifying the Problem: Preparing for the FCS Workshop

The workshop was moderated by two researchers from CanChild and one researcher from Brazil, who are experienced in developing and implementing FCS. The co-founders of *Nossa Casa Institute* organized two online meetings with the researchers to discuss the workshop format from their previous experience and the relevant content related to the FCS literature. The workshop audience included 16 families of children and adolescents with disabilities, 2 adults with CP, 40 health care professionals, 10 healthcare service coordinators, and 16 academics (i.e., professors, researchers, and students).

The structure of the online workshop included access to pre-recorded educational videos and interactive online sessions. The pre-recorded videos presented preliminary information for the online discussions, as well as providing the participants access to the main literature regarding FCS. Participants were encouraged to access the educational videos prior to each online interaction to create a common starting point for discussions. The online interactions were designed to promote opportunities for all participants actively to build competencies in FCS. Each interaction lasted 1 h on average. The workshop had 3 modules: Module 1: “Challenges and barriers to incorporate family-centered principles”; Module 2: “What is my contribution to family-centered service?” and Module 3: “What can we do together?” The modules were organized so that the participants could share initial thoughts of challenges regarding the FCS principles and then build their own competencies and possibilities to assume family-centered behaviors and attitudes.

### Adaptation of the Knowledge to the Local Context: Module 1

The workshop started with an online interaction to present a historical perspective of FCS for children with disabilities and debate its main principles. For that purpose, CanChild researchers discussed the main principles and a brief historical perspective of FCS. All participants were encouraged to post their questions and considerations in an online chat to show their opinions and comments. Such interaction facilitated an understanding of the main concerns and expectations of the participants. Three pre-recorded educational videos were available with the main characteristics of FCS.

### Assessments of Barriers to Knowledge Use: Module 1

After the first online session, we sent an anonymous online survey to be completed by the participants, built specifically for the workshop. We prepared five surveys following the same structure, but including information specific to the group of the participants (i.e., adults with CP, families of children/adolescents with disabilities, health care professionals, health care coordinators, and academics). In this survey, we asked opinions of the people regarding the main barriers to the implementation of FCS principles in Brazil, and the challenges they faced. We were interested in issues and experience with the services undergone by the children and families. We wanted to hear the experiences of adults with CP in their daily routines and services, and in their interactions with health care professionals, coordinators, and researchers. It was important to learn which FCS principles are already incorporated in the care of their child (families), the care of adults with CP in their daily practices and services with health care professionals, coordinators, and researchers, and which aspects related to FCS they would like to discuss in the workshop.

The information from the online survey of the participants was classified according to challenges related to the behaviors, attitudes, and actions of therapists; behaviors and expectations of families; and the actions of those involved in the format and regulations of services in Brazil. The main concerns reported by the participants were discussed in a second online live interaction to elucidate the possible myths and common misunderstandings underlying FCS.

### Selection and Implementation of Interventions: Module 2

Module 2 was designed to discuss strategies to improve the implementation of principles of FCS. We prepared six pre-recorded video lectures in Portuguese (Brazil) based on FCS sheets of CanChild (28): effective communication; building on competencies of families; respect; negotiation; partnership; and decision-making process. The choice for these topics was based on the challenges and barriers that participants reported in the survey. We provided two additional lectures with instrumental information related to FCS implementation: assessment tools to measure FCS outcomes and analysis of family involvement in interventions for children with CP.

The online interactions occurred in four main groups: families and adults with CP, health care professionals, health care coordinators of services, and academics (i.e., professors, undergraduate, and graduate students). In these separate meetings, each group was assigned a specific topic for discussion, with the assistance of a moderator. With families and adults with CP, we asked the participants to discuss their main priorities and needs from rehabilitation services and health care professionals, based on FCS principles. Health care professionals discussed how to improve the family-centered relational components (e.g., behaviors, attitudes, and values) and operational components (i.e., assessment tools and intervention strategies). Health care coordinators discussed ideas to structure their services in light of family-centered characteristics. Academics, including professors, graduate, and undergraduate students, were asked to consider the academic role in implementing FCS.

### Monitor Knowledge Use: Module 2

After discussing their main ideas, the participants in each group were asked to prepare a small presentation to be shared in the last meeting. Their interactions had previously been moderated by the Brazilian researcher in Telegram discussion forums.

### Evaluate Outcomes: Module 3

In the last online interaction, the groups presented their ideas and suggestions from the separate discussions. All participants were encouraged to present their perspectives to identify effective strategies to promote FCS practices. The first group to present were the “families and adults with CP group,” who expressed their needs and their expectations from services and therapists. Parents reinforced their desire to be listened to and trusted by therapists regarding their priorities; they asked for their voices of children to be heard; they believe that their children should be seen as children who need time to play and to have fun; and they reported the desire that therapists should involve not only mothers but also fathers and the extended family in their rehabilitation program. They were also encouraged to express their opinions during the presentations of the other groups. The following three groups (health care professionals, academics, health care providers) presented relational and instrumental strategies they recommended to be adopted in their daily practices. After their presentations, we asked the participants to report their comments and suggestions about their experience at the workshop.

### Sustain Knowledge Use: After the FCS Workshop

After the workshop, we invited the participants to join one Telegram group. In this group, they were able to contact each other and share experiences. The information regarding the feedback of the participants in the workshop helped to create online information on the social media channels from *Nossa Casa Institute*. In addition, such information supported the planning and development of an online conference (Online CP Conference: From all to All), aimed at improving the dissemination of knowledge among individuals with CP, families, health care professionals, and researchers. Specific information regarding the online conference and its impact will be reported in future studies.

## Lessons Learned

*Nossa Casa Institute* was conceived to share information about the daily living of individuals with disabilities, with emphasis on CP. It is also creating opportunities for listening and exchanging knowledge through actions on social networks (e.g., interactive video lives, campaigns, and educational workshops) with the participation of all stakeholders. The involvement of families and individuals with disabilities in activities held at *Nossa Casa Institute* has contributed to the greater empowerment of these populations. Furthermore, these actions are enhancing the awareness and education of health care professionals and researchers and reinforcing the value of involving families and individuals with disabilities in research and educational initiatives.

One of the main challenges experienced by the *Nossa Casa Institute* is related to the lack of financial support from the Brazilian government or other agencies; so far, such activities are conducted by volunteering work from its collaborators. Possible subsidies would support future KT activities at *Nossa Casa Institute*. Future studies are planned to explore and evaluate the impact of the proposed actions to support the KT in the pediatric rehabilitation field in Brazil.

## Data Availability Statement

The raw data supporting the conclusions of this article will be made available by the authors, without undue reservation.

## Author Contributions

MB coordinated the project and was involved in the conception, writing, and review of the manuscript. MA, BV, and MM were involved in in the conception, writing, and review of the manuscript. DC and RB are representatives of families and adults with cerebral palsy and they helped in the conception and review of the manuscript. RT and PR were involved in the conception and review of the manuscript. All authors contributed to the article and approved the submitted version.

## Conflict of Interest

The authors declare that the research was conducted in the absence of any commercial or financial relationships that could be construed as a potential conflict of interest.
